# A Practical Guide to Translating Scientific Publications Into Aphasia-Friendly Summaries

**DOI:** 10.1044/2025_persp-24-00141

**Published:** 2025-04-04

**Authors:** Anna V. Kasdan, Deborah F. Levy, Isaac Pedisich, Stephen M. Wilson, Dominique Herrington

**Affiliations:** a Department of Hearing and Speech Sciences, Vanderbilt University Medical Center, Nashville, TN; b Princeton Writing Program, Princeton University, NJ; c Independent Researcher, Philadelphia, PA; d School of Health and Rehabilitation Sciences, University of Queensland, Brisbane QLD

## Abstract

**Purpose:**

Research in aphasiology is largely not accessible. Almost none of the articles published in the field’s rich, over 150-year history are communicated in a way that is understandable to those who could benefit from them the most—individuals with aphasia and their loved ones. In this tutorial, we detail how researchers in any field of aphasiology can create aphasia-friendly research summaries of their scientific publications. This step-by-step guide in eight simple parts covers principles of aphasia-friendly written communication (e.g., use of plain language and supportive icons and images) and makes use of freely available resources. We also introduce a prototype tool—Article Friend—that automatically generates aphasia-friendly abstracts to jump-start this process for researchers; this preliminary tool serves as a proof of concept that creating accessible research can be an efficient, sustainable practice in the scientific publishing landscape.

**Conclusions:**

The tutorial provides researchers with specific tools and examples to effectively and easily create aphasia-friendly summaries of their publications. Principles from our tutorial extend beyond aphasia and can apply to consumers affected by other communication and cognitive disorders, such as developmental language disorder, dementia, and traumatic brain injury. Making research available to patient stakeholders and their loved ones can empower them to access and understand the research they have contributed to, ultimately furthering increased community engagement and interchange between researchers, clinicians, consumers with aphasia, and policymakers.

**Supplemental Material:**

https://doi.org/10.23641/asha.28590227

Aphasia-friendly materials are translations of written materials that aid comprehension for people with aphasia. Such modifications include simplified vocabulary and syntax, use of keywords, large and standard font, supportive graphics and icons, and increased white space on the page (Brennan et al., 2005; Herbert et al., 2012; Rose et al., 2003). Aphasia-friendly materials are a staple in clinical and community group settings for helping individuals communicate with those around them. Research on such materials has been ongoing for decades; here, we highlight a more recent portion of this work given the brief and practical focus of this tutorial. In small experimental studies, people with aphasia have demonstrated improved comprehension of medication instructions (Saylor et al., 2022), health information (Rose et al., 2003), and written paragraphs (Brennan et al., 2005) with aphasia-friendly modifications. These studies demonstrate the importance of aphasia-friendly materials in supporting comprehension and independence for people with aphasia as they access complex information (Worrall et al., 2005).

Despite the prevalence of aphasia-friendly tools in clinical settings, they are strikingly absent in research settings. A search for “aphasia” on PubMed as of October 7, 2024, yields over 21,000 results; almost none of these are accompanied by accessible versions. This highlights that the vast majority of people with aphasia and their loved ones lack important knowledge pertaining to the research being done for and about them (Brady et al., 2013), specifically in the area of treatment research (Hinckley et al., 2014), despite people’s strong desires to be involved in research and have access to information (Dalemans et al., 2009; Finch et al., 2023; Rose et al., 2010). Additionally, accessible research materials can facilitate interest and participation in research studies for individuals with aphasia (Pearl & Cruice, 2017).

Some journals offer the opportunity to accompany scientific papers with aphasia-friendly materials such as plain language summaries, video summaries, and graphical abstracts alongside publications (Hinckley & ElKhouri, 2023; King et al., 2023; Pierce, 2023). In our experience, even journals that do not explicitly have these alternative publication practices are willing and able to publish such accompaniments in their supplemental materials (Casilio et al., 2025; Levy et al., 2022, 2024). Disseminating research in these accessible formats allows people with aphasia and their loved ones to learn about the research they contributed to and better advocate for themselves. Accessible research practices can promote diversity, equity, and inclusion in publishing (Rosenberg et al., 2023) and may, in large-scale public health and epidemiological contexts, aid in addressing health disparities (Rios et al., 2016).

There are some resources and publications already available about creating accessible aphasia-friendly content. While these resources are extremely useful, they either do not focus on translations of scientific research articles specifically—instead focusing on information about services or explanations of care (Herbert et al., 2012, 2019)—or do not directly address precisely *how* to create accessible research summaries (Hinckley & El-Khouri, 2023)—for example, in where to find supportive images or how much text should correspond to each section of a traditional scientific manuscript. In this tutorial, we provide specific examples of full translations of research summaries that have been published in peer-reviewed journals as created by our group; to our knowledge, extant research articles about disseminating aphasia-friendly research have not included such tangible accompaniments.

This manuscript presents a detailed “how-to” for researchers interested in creating aphasia-friendly versions of their manuscripts, to complement traditional scientific publications. Our tutorial is targeted toward any researcher in the field of aphasiology—or indeed, research relevant to individuals with brain or communication disorders in general—regardless of specific discipline (speech-language pathology, cognitive neuroscience, linguistics, etc.).

## The Tutorial

In this section, we present a step-by-step “how-to” guide for creating accessible, aphasia-friendly research summaries. The tutorial is divided into eight parts, with succinct instructions for each part. Our tutorial combines general principles for presenting content (e.g., plain language) with our own recipe for formatting aphasiafriendly summaries (e.g., using Google Slides). Of course, there are other possibilities for going about this process and interested authors are free to customize certain aspects of the tutorial for their own needs. This section simply provides one clear path forward for generating accessible research summaries.

A translated research summary should succinctly answer the following questions:

1. Why did the authors do this research in the first place?
2. What did the authors do?
3. What did the authors find?
4. Why are the findings important for people with aphasia and their loved ones?

**Figure 1.**
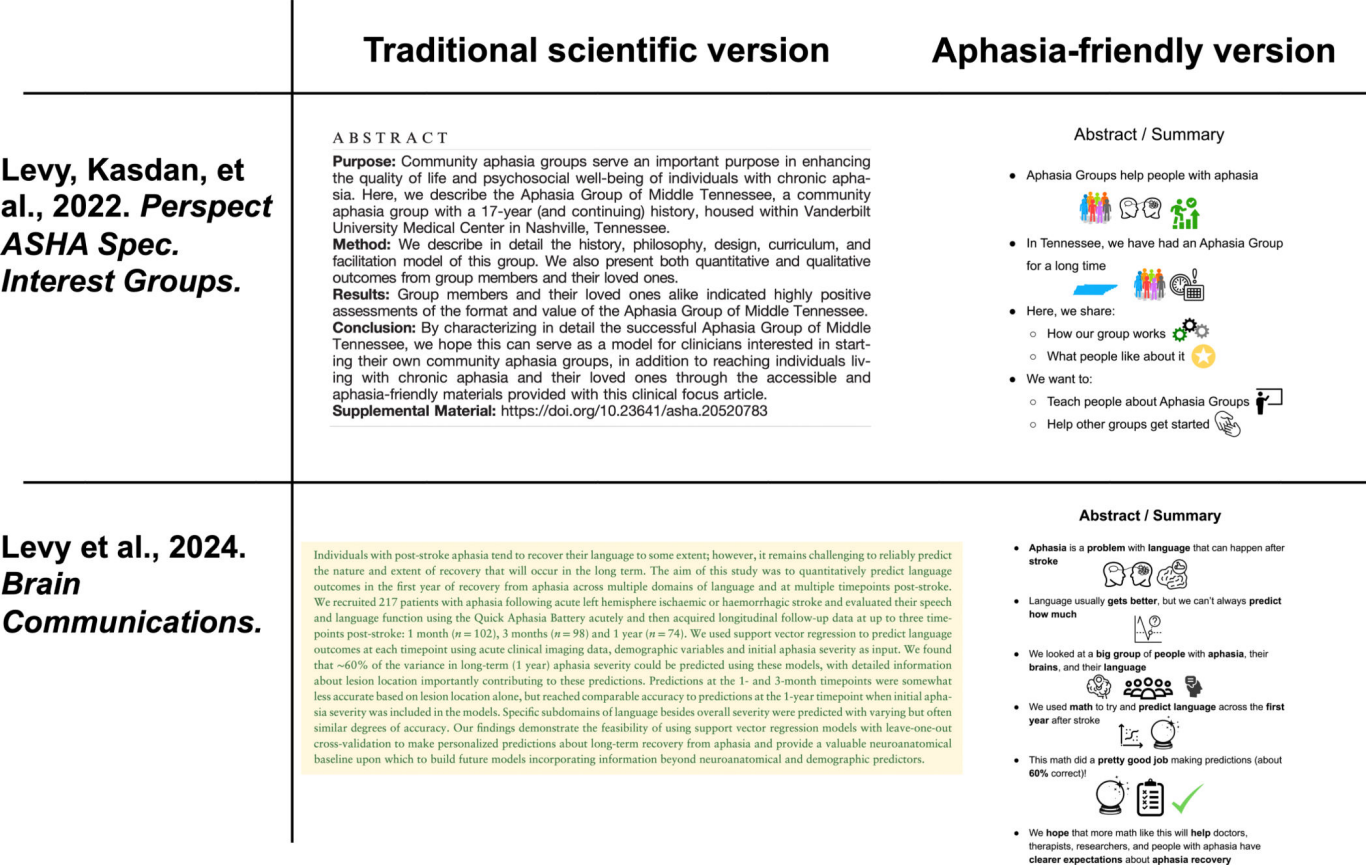
Comparison of traditionally written abstracts and their aphasia-friendly translations for two key publications from our group. Aphasia-friendly abstracts incorporate plain language, bolding of key words, and supportive icons.

When following the suggestions below, researchers may use our prior publications (Levy et al., 2022, 2024) as examples of aphasia-friendly research translations. These publications provide two concrete examples of how research across disciplines and methodologies in aphasia (one, surveys about community groups, and the other, a machine learning approach to modeling neural data to predict language outcomes) can be translated into aphasiafriendly formats. Members of the Aphasia Group of Middle Tennessee indicated preference, in an informal community group discussion, for the aphasia-friendly version of Levy et al. (2022), compared to the traditional text version; these sentiments were echoed by community members with aphasia at the 2023 Aphasia Access Summit. Figure 1 shows a side-by-side comparison of the traditional, scientific abstracts and the aphasia-friendly versions, created per the guidelines of this tutorial, for both articles. The full, published aphasia-friendly manuscripts for both articles are included in the Supplemental Materials.

**Example 1:** Levy et al. (2022); see Supplemental Material S1.

**Example 2:** Levy et al. (2024); see Supplemental Material S2.

### Step-by-Step Tutorial in Eight Simple Parts

Formatting instructions are for Google Slides because it is freely available; however, researchers may use any slide program of their choice (more in the “Application Choice” section below).

#### Part 1: Plain Language

- Write in plain language using the Federal Plain Language Guidelines (2011, plainlanguage.gov). An abbreviated version of these recommendations is included below and can also be found in Hinckley and El-Khouri (2023). Characteristics/guidelines include the following:
  - Short sentences
  - Simplified syntax (all sentences in the active voice)
  - Common, frequent words
  - Words with few syllables
  - Avoid double negatives
  - Avoid scientific jargon. If a scientific term must be used, define in plain language.
- Write at approximately a sixth-grade reading level according to a Flesch–Kincaid calculator (https://goodcalculators.com/flesch-kincaid-calculator/); using readability formulas such as Flesch–Kincaid can aid in reducing readability levels and consequently improve understanding of health-related information for people with aphasia (Aleligay et al., 2008; Ley & Florio, 1996).

#### Part 2: Aphasia-friendly Formatting

- Bold key words.
- Use large font.
- Use ample white space and do not crowd texts and images.
- Use a white background with black, standard sans serif font.
- Minimize multiline statements.
  - Long sentences can be broken up using ellipses or sub-bullets (cf. the above examples).
- Additional recommendations on formatting can be found in Herbert et al. (2012).

#### Part 3: Supportive Icons and Images

- Images should represent a key word (content nouns or verbs) in a sentence to aid in comprehension.
- Images should directly support the text and not be superfluous.
- Approximately one image should accompany at least every other bullet point (but see the Discussion section for more details on this point).
- Use clear and simple images that are not infantilizing or stigmatizing (e.g., not clip art of groups of children when referencing a group of people).
- Color should primarily be used when it coveys meaning (Herbert et al., 2019).
- Images must be licensed to be published and reproduced or be in the public domain. Resources for finding images include:
  - Free
    1. Google Images search. Tools → Usage rights → Creative Commons licenses (this *must* be selected!). These options appear after you search for an image, and not before.
    2. Microsoft PowerPoint search. Insert → Pictures → Online Pictures (make sure “Creative Commons only” is selected).
    3. Wikimedia Commons (https://commons.wikimedia.org/wiki/Category:Images)
    4. Openclipart (https://openclipart.org/)
    5. ParticiPics (https://www.aphasia.ca/participics/). This is a free image repository designed specifically for communicating with people with aphasia. The repository is hosted by the Aphasia Institute. Users will need to register for a free account to download images.
  - Paid
  1. Noun Project (https://thenounproject.com/)
  2. BioRender (https://www.biorender.com/)
  3. Shutterstock (https://www.shutterstock.com/)

This is a nonexhaustive list of image resources. It is imperative that authors check the licensing agreements on all images they plan to reproduce in published work; authors must make attributions where necessary.

#### Part 4: Application Choice

- Google Slides (or PowerPoint, Keynote). Slide applications are preferred because they:
  - Offer superior and more flexible layout opportunities compared to word processors, especially when including graphics
  - Encourage users to think intentionally about one section at a time, rather than working through a continuous document without delineated sections.

#### Part 5: Document Setup

- In Google Slides ...
  1. File → Page Setup → Custom → 8 × 11.5 in. or A4 (210 × 297 mm)
  2. Slide → Apply layout → Title and body

#### Part 6: Manuscript Sections

- Title
  - Top text title: a simplified and shortened version of the original title, similar to a running head (size 28 font).
  - Middle: representative images from the paper that summarize the “main idea.”
  - Insert two text boxes beneath images
    1. Middle text: list of authors (size 14 font).
    2. Bottom text: write “An accessible version of:” followed by the manuscript’s original title (size 14 font).
- Abstract
  - A few bullet points (max: 6) that can stand alone as a summary of the research; a good rule of thumb may be to convert each sentence of the original abstract into a single bullet point in the aphasia-friendly document.
  - The abstract should be geared toward individuals with aphasia and their loved ones (i.e., What would a person with aphasia want to get out of reading this research paper? Why should they care?).
- Main text
  - As in the original manuscript, sections should include Introduction, Methods, Results, and Discussion.
  - Start each page with a short title (see examples), followed by text summarized in bullet format. Titles may be the name of the section (e.g., Introduction), a summative statement (e.g., Writing is important.) or question (e.g., Why is writing important?).
  - Each section should be summarized in two to three pages with no more than five bullet points per page.
  - Main ideas should be delineated with solid black bullets and steps/intermediary points with sub-bullets.
  - Translate the information from the original manuscript that is most essential to convey to people with aphasia. It may be that an extensive summary of prior literature, although often present in academic manuscripts, is less important to describe to people with aphasia.
- Conclusion
  - A few bullet points (max: 4) that recapitulate, in plain terms, the take-home messages of the research. Similar to the abstract, this page should stand alone as a summary of the research.
  - The summary should emphasize what the research means for people with aphasia and their loved ones. For example, perhaps there is an emphasis on future clinical implications, rather than how the research contributes to a theoretical framework in the field.

#### Part 7: Document Review

- Authors should review the document to ensure that all text is in plain language and no higher than a sixth-grade reading level according to the Flesch–Kincaid calculator.
- Images should be aesthetically cohesive throughout the document (e.g., using the same or similar icons across sections to convey a repeated point).
- Bolded key words should ultimately stand alone (i.e., reading only the bolded words should convey a high-level summary of the research).
- Export the document. File → Download → PDF Document (.pdf).

#### Part 8: Stakeholder Feedback

- Authors should ideally aim to share their summary with an individual or a small group of people with aphasia prior to publication.
- Areas for feedback may include clarity of the summarized research findings, use of supportive images, and length of the summary.
- Involving consumers themselves in the research process aligns with recommendations for aphasia research video abstracts, where people with aphasia want to be included in the process (Finch et al., 2023; Pierce, 2023).

## Future Directions

### A Proof-of-Concept Online Tool: Article Friend

While developing this publication, we made a prototype version of a new, free, online tool—Article Friend—that automatically generates accessible research abstracts with the aid of the large language model ChatGPT (OpenAI, 2024); abstracts are to be subsequently refined by researchers. Article Friend is a proof of concept and is still being optimized; at the time of publication of this manuscript, this prototype produces aphasia-friendly research abstracts only. However, such abstracts may be useful as a means of condensing the most important ideas from an author’s research into an aphasia-friendly format. Users may copy and paste the text of a scientific abstract into our web tool that will then auto-generate an aphasia-friendly version of that abstract (e.g., bolded content words, supportive icons and images, bulleted text) in Google Docs format. Article Friend is a good starting place for authors looking to begin the process of creating accessible manuscripts; it is user-friendly and accessible. This beta tool calls prompts to ChatGPT such as “search terms should only be very common words” and “avoid using any words that have homonyms—for example, never use the word ‘change’ because it might mean ‘affect’ or ‘money’”; these closely mirror the guidelines presented above for creating aphasia-friendly summaries “by hand.” As Article Friend continues to be developed and refined, it can be highly customized to individual research teams’ needs.

Future work may (a) expand this tool to generate fulllength aphasia-friendly research summaries, (b) incorporate consumer feedback on the quality and effectiveness of abstracts produced with this new tool, and (c) include specific types of supports to target different levels of aphasia severity. While the level of abstract summaries currently generated from Article Friend may not be a “one size fits all” for all individuals with aphasia and their communication needs, this tool is an important step forward in accessibility. Abstract summaries based on Levy et al. (2022, 2024) created using Article Friend in October 2024 are available in Supplemental Materials S3 and S4 (“Helpful Aphasia Group at Vanderbilt” and “Predicting Speech Recovery After Stroke,” respectively).

A link to Article Friend is available: http://www.article-friend.com. Note that our intention is exclusively for this tool, as it currently stands, to be used *by researchers*, and not yet for public use. Given the proliferation of misinformation following the introduction of large language models into the mainstream (De Angelis et al., 2023), it is imperative that expert researchers check and edit the output of these tools prior to publication. In a similar ethical vein, any uses of this and/or related large language models during the research process must be explicitly disclosed.

## Discussion

We have presented a step-by-step tutorial for academic researchers interested in creating aphasia-friendly research summaries of their scientific manuscripts. Our work adds to the body of literature on this topic by providing a comprehensive set of resources and recommendations, accompanied by concrete examples of what these research summaries look like when published in peer-reviewed journals. Individuals with aphasia and their loved ones—those who may benefit from research the most—deserve opportunities to access and feel empowered to understand the research they have contributed to. The tools we have provided here will allow researchers to efficiently and effectively make this accessibility a reality.

It is our hope that the core tenet of this paper—communication of accessible research—extends beyond the realm of aphasia specifically, to communication disorders populations at large. Patients and their families who are directly impacted by both future *directions* of research and research findings deserve to have research communicated to them in a straightforward way. Many of the principles we draw on for creating materials accessible to the aphasia community can apply to science communication efforts at large (e.g., Federal Plain Language Guidelines; Edgell & Rosenberg, 2022; Rosenberg et al., 2023) and have been suggested by other researchers in the field (e.g., Herbert et al., 2012; Hinckley & El-Khouri, 2023). Guidelines for creating accessible research summaries for other communities with communication differences, including those with developmental language disorder (DLD) have been developed (Gasparini et al., 2024); each target audience benefits from their own specific set of modifications. We hope that it will be become the norm to publish guidelines tailored to specific populations, including traumatic brain injury and dementia populations who have been involved in other aspects of the research lifecycle (Bethell et al., 2018; Ziaya et al., 2016), and that researchers will use these guidelines early and often in the publication process.

While the authors of a study may be best situated to develop aphasia-friendly versions of their scientific papers, there are other possibilities as well. Creating such summaries would be a phenomenal educational opportunity for undergraduate or speech-language pathology students to hone their skills in communicating scientific research to groups with communication disorders. Students could create aphasia-friendly versions of seminal, already published papers in aphasiology to ensure people with aphasia have access to the most influential research in the field, in addition to ongoing and newly published research. Another promising direction for creating these summaries is using ChatGPT or other large language models to jump-start the process, as we have done in our prototype, Article Friend. Related work with image generation for aphasia assessment is being done using DALL-E 2 (Pierce, 2024). Note, however, that these online tools should always be used in conjunction with researcher expertise, and not as a stand-alone method.

Future work in this area could also explore which aspects of aphasia-friendly modifications are most beneficial for comprehension and which tools and recommendations are best suited for different levels of aphasia severity. While we recommend using one image for at least every other bullet point in an aphasia-friendly research summary, future empirical research is needed to determine the best image frequency for supporting understanding of the written text. Some work suggests that images and icons do not significantly aid in comprehension (Brennan et al., 2005; Wilson & Read, 2016), especially when the images are not personalized (Wallace et al., 2012). There are many design considerations (e.g., image layout, image context) that may influence how useful visuographics can be in supporting understanding for people with aphasia (Brown & Thiessen, 2018) and patient populations more generally (Hoffmann & Worrall, 2004). People with aphasia may benefit from being involved in the design process of aphasia-friendly materials (Herbert et al., 2019) given their preferences for and perceptions of different aspects of these modifications (Dietz et al., 2009; Rose et al., 2012).

Critically, we need to consider how to disseminate aphasia-friendly research summaries to individuals with aphasia, considering that most research is still published under paid subscription services. One option is to incorporate them as supplementary material with the articles themselves. This could certainly be effective, especially if the paper is published open access, bearing in mind that few members of the public will be able to access paywalled scientific content. Another option is to use open data sharing platforms such as Open Science Framework, which allow researchers to disseminate research free of charge to the larger community. Even with open-access options, stakeholders may struggle to navigate these platforms designed for individuals with knowledge about research and publishing.

One solution to these issues of publication access could be to disseminate aphasia-friendly research summaries to community members directly. The nonprofit organization National Aphasia Association (NAA, 2024) has an active website (aphasia.org) that people with aphasia and their loved ones directly engage with. Since many individuals with aphasia are likely already familiar with and use the NAA website for other resources, posting research summaries there would limit barriers to access. Similar strategies could be used to disseminate materials to aphasia centers in the United States (e.g., Brooks Rehabilitation Aphasia Center in Jacksonville, FL; Aphasia Center of California in Oakland, CA) and to local aphasia organizations. Moreover, the broader principle of integrating accessible research into resources patient stakeholders are already aware of can be extended to other populations (e.g., integrating research accessible to the DLD community with DLD [2024] and Me; dldandme.org). None of the abovementioned strategies are mutually exclusive: Aphasia-friendly summaries could be included as supplemental materials with journal articles, posted in advance to open-access repositories to ensure free access, and shared via communities such as the NAA.

Finally, aphasia-friendly summaries can also facilitate community engagement between researchers, clinicians, patients, and care partners/loved ones. For example, aphasia-friendly summaries could benefit clinicians looking to access research on aphasia therapies and treatments. Typically, clinicians have only limited time to delve into outside reading and comb through the hundreds of relevant papers published every month. Clinicians could use these summaries as one tool in their toolbox for accessing and interacting with research, as even academic paper titles and abstracts, though brief, can contain significant scientific jargon (Martínez & Mammola, 2021). Paid subscription services such as The Informed SLP (2025; https://www.theinformedslp.com/) provide plain language summaries of the most clinically relevant scientific journal articles. If academic researchers start to systematically disseminate accessible research summaries, this could aid to bridge the notoriously stagnant research-to-practice gap (Douglas et al., 2023). Another example of engagement would be to incorporate aphasia-friendly summaries into speech therapy sessions, either as stimuli in therapies such as Multiple Oral Re-Reading (Moyer, 1979) or Attentive Reading with Constrained Summarization (Rogalski & Edmonds, 2008) or as general discussion and rapport-building material with patients and their loved ones, w ho may be present at sessions. Such accessible materials c ould help to build trust between patients, clinicians, and researchers if patients begin to develop an understanding of how their participation in research can benefit science and others like them.

Our tutorial on creating aphasia-friendly research summaries is one step toward advancing scientific communication for individuals with communication disorders and influencing policies that support accessibility of science and health information. England’s Accessible Information Standard (DCB1605), first effective in 2016, establishes legal guidelines for making health and social care information accessible and understandable to patients and their care partners. We hope that academic researchers will incorporate accessible research summaries into their work, never losing sight of whom their research can most directly impact—people with aphasia and their communities.

## Supplementary Material

Supplemental Material S3

Supplemental Material S4

Supplemental Material S2

Supplemental Material S1

## Data Availability

Data sharing is not applicable to this article as no new data were created or analyzed in this tutorial.
