# Supplementary material for: A Practical Guide to Translating Scientific Publications Into Aphasia-Friendly Summaries
Source: Perspect ASHA Spec Interest Groups. Author manuscript; Available in PMC 2025 Jun 30. (PMC12208688; doi:10.1044/2025_persp-24-00141)

Helpful Aphasia Group at Vanderbilt

Authors: Deborah F Levy, Anna V Kasdan, Katherine M Bryan, Stephen M Wilson, Michael de Riesthal, Dominique P Herrington

An accessible version of:

Designing and Implementing a Community Aphasia Group: An Illustrative Case Study of the Aphasia Group of Middle Tennessee

The **Aphasia Group** helps people with chronic aphasia.

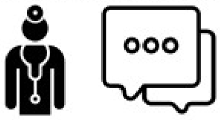

The group is part of **Vanderbilt University** in Nashville.

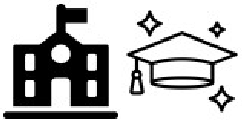

It has been running for **17 years**.

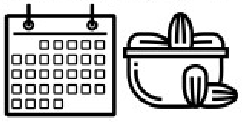

The group's methods and results are shared.

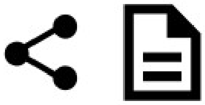

Members and their families **like** the group a lot.

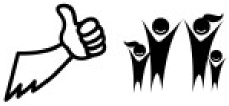

This group could guide **new aphasia groups**.

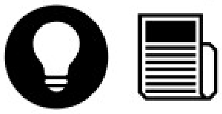

Supplement: Supplementary file 1 [file NIHMS2072786-supplement-Supplemental_Material_S1.docx]
