# Supplementary material for: A Practical Guide to Translating Scientific Publications Into Aphasia-Friendly Summaries
Source: Perspect ASHA Spec Interest Groups. Author manuscript; Available in PMC 2025 Jun 30. (PMC12208688; doi:10.1044/2025_persp-24-00141)

Predicting Speech Recovery After Stroke

Authors: Deborah F Levy, Jillian L Entrup, Sarah M Schneck, Caitlin F Onuscheck, Maysaa Rahman, Anna Kasdan, Marianne Casilio, Emma Willey, L Taylor Davis, Michael de Riesthal, Howard S Kirshner, Stephen M Wilson

An accessible version of:

Multivariate lesion symptom mapping for predicting trajectories of recovery from aphasia

This study looked at **speech recovery** after a **stroke**.

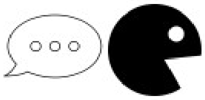

Researchers tested **217 patients** with **aphasia**, a language problem.

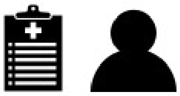

Three tests were done: right away, at 1 month, 3 months, and 1 year.

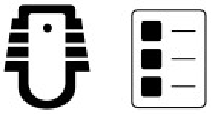

They used a math model with **brain images** and personal details to guess recovery.

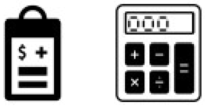

The model could guess recovery after 1 year about **60% right**.

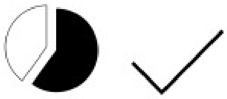

The guesses got better when they included how bad the **aphasia** was at first.

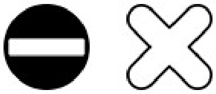

Overall, the method could help guess how well a person might get their language back.

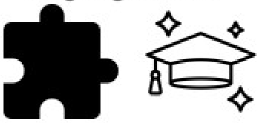

Supplement: Supplementary file 2 [file NIHMS2072786-supplement-Supplemental_Material_S2.docx]
